# Understanding the Emotional Impact of GIFs on Instagram through Consumer Neuroscience

**DOI:** 10.3390/bs11080108

**Published:** 2021-07-30

**Authors:** Idoia Rúa-Hidalgo, Maria Galmes-Cerezo, Carmen Cristofol-Rodríguez, Irene Aliagas

**Affiliations:** 1 Faculty of Business and Communication, International University of La Rioja, 26006 Logroño, Spain; idoia.rua@gmail.com (I.R.-H.); irene.aliagas@unir.net (I.A.); 2 Departament of Organization and Marketing, Faculty of Economics & Business, Somosaguas Campus, Universidad Complutense de Madrid, 28223 Madrid, Spain; mgalmes@ucm.es

**Keywords:** social networks, digital consumer behavior, emotion, Instagram, GIF, consumer neuroscience, neuromarketing, skin conductance, facial coding, eye tracking, sentiment analysis

## Abstract

The ability of GIFs to generate emotionality in social media marketing strategies is analyzed. The aim of this work is to show how neuroscience research techniques can be integrated into the analysis of emotions, improving the results and helping to guide actions in social networks. This research is structured in two phases: an experimental study using automated biometric analysis (facial coding, GSR and eye tracking) and an analysis of declared feelings in the comments of Instagram users. Explicit valence, type of emotion, length of comment and proportion of emojis are extracted. The results indicate that the explicit measure of emotional valence shows a higher and more positive emotional level than the implicit one. This difference is influenced differently by the engagement and the proportion of emojis in the comment. A further step has been taken in the measurement of user emotionality in social media campaigns, including not only content analysis, but also providing new insights thanks to neuromarketing.

## 1. Introduction

Currently, social media marketing strategies seek to position brands within the hearts of their customers, where the main experience of value is emotion [1,2] (Smith and Bolton, 2002; Mauri, et al., 2011). This is why the involvement of the senses is fundamental to influencing the emotional state of social media users [3] (Prescott, 2017). One of the most widely used resources to achieve this emotional impact on users is GIFs. Their effectiveness has been analyzed in several studies that have shown their ability to generate emotionality [4,5,6] (Bourlai and Herring, 2014; Bakhshi et al., 2016; Gygli and Soleymani, 2016).

In fact, nowadays, communication and marketing professionals are looking for tools that allow them to measure the effectiveness of their campaigns, in terms of emotionality. The most frequently used research techniques are based on content analyses of comments made by a brand’s followers on a social network [7,8,9,10] (Driscoll, 2015; Turnbull and Jenkins, 2016; Scheinbaum, 2017; Kim and Kim, 2018). Less common is the use of neuroscience techniques to measure emotionality based on users’ unconscious responses to a given stimulus.

Consequently, the present study proposes a combination of emotion measures (emotional valence, basic emotions and engagement) to assess the effectiveness of GIFs as generators of emotional experiences on social networks. In addition, neuroscience techniques were used to observe physiological and cognitive responses (implicit measures), as well as to perform sentiment analyses (explicit measures) of Instagram comments.

## 2. Literature Background

### 2.1. Conceptualization of Emotion Assessment

There are a large number of definitions of the concept of emotion in existent literature, as well as of existing emotional states, the ways to measure them, and their neurophysiological representations. In this investigation, emotion is understood as the cognitive process of evaluating and interpreting feelings, with the aim of regulating social and/or relational responses in social networks. To address emotion, there are two traditional measurement perspectives in the field of psychology. The first is the dimensional measurement of emotion, which states that an emotion is composed of valence and arousal. Emotional valence is the positive or negative evaluation of the emotional state, while arousal (The construct “arousal” is a hypothetical term that describes the processes that control alertness, wakefulness and activation. (Anderson, 1990) [11] or physiological arousal refers to the activation of the parasympathetic nervous system (e.g., increased skin sweating or heart rate) [12,13,14] (Harmon-Jones et al., 2017; Izard, 2010; Lang, 1995). The second is the measurement of emotion as a discrete entity. In this case, the emotional evaluation process results in concrete emotions, such as happiness or sadness [12] (Harmon-Jones et al., 2017). Specifically, six basic and universal emotions have been identified: happiness, surprise, fear, anger, disgust and sadness [15] (Ekman, 1993).

Therefore, this emotionality underlies psychological and physiological responses [12] (Harmon-Jones et al., 2017). This is stated since emotions are normally triggered by a stimulus that is perceived or remembered, provoking physiological actions, such as the contractions of certain facial muscles [16] (Damasio and Carvalho, 2013). Moreover, authors such as LeDoux and Brown (2017) [17] have investigated which brain circuits activate a specific emotion and allow us to be aware of it and express it verbally. Consequently, it is necessary to combine its explicit (textual) study with tools that allow us to capture the most implicit part of the emotion (neuroscientific and biometric tools).

### 2.2. Sentiment Analysis and Emotional Engagement in Social Media

Several studies have been devoted to the analysis of sentiment in social networks. Among the most relevant is that of Driscoll B. (2015) [7], who studied sentiment in 20,189 tweets and 921 replies, concluding that 38% of these replies express a positive emotion while 20% express a negative emotion. This also highlights the importance of emotion and its link to perceived intimacy between senders and receivers. For Turnbull and Jenkins (2016) [8], social media reactions offer marketers the opportunity to better understand how consumers engage emotionally with social media content, enabling greater precision in their emotional response. This allows brands to more effectively measure their campaigns.

Social media, and Instagram in particular, are experiential products that continuously reinforce both positive and negative habits [9] (Scheinbaum, 2017). Several studies point to the bias of this online positivity, as most content distributed on social networks is rated more positively than negatively [18,19] (Reinecke and Trepte, 2014; Waterloo, Baumgartner, Peter and Valkenburg, 2018). In the context of emotions and positivity on the internet, there is research that advocates the expression of emotions through networks, from which a direct link between emotional language and online behavior can be inferred [20,21] (Dresner and Herring, 2010; Huffaker, 2010).

Following this last idea, the link between behavior and expressed emotion has been seen in studies that have investigated the emotional state and the engagement in social networks. Most notably, Dubovi and Tabac (2021) [22] have tried to determine whether the behavioral engagement of views, likes, dislikes and comments, and the emotional and cognitive engagement in science dissemination channels on YouTube, coincide or not. They show in their study that, regardless of the valence of emotional engagement, emotion is linked to higher behavioral engagement in posting comments and to higher cognitive engagement in argumentative deliberation. Morgado et al. (2020) [23] studied the emotional engagement of users on the police’s Facebook profile, concluding that the overall engagement is positive and that it mainly came from women. In contrast, Vizcaíno and Aguaded (2020) [24] study the emotional poralization of children on Instagram accounts. Their results reveal a prominent positivity and subjectivity in the lexical field, with the repeated use of adjectives such as “happy”, “new” or “super”. On the other hand, Kim and Kim (2018) [10] conducted explorations on computer vision techniques on Instagram to define associations between personality and gender by means of photography. Their results show that users’ extroversion, agreeableness and openness were partly associated with the emotions expressed in faces in their photos, specifically among certain pixel traits. It was also observed that the big five personality traits can be predicted by the above variables, except in the case of gender. Claffey and Brady (2019) [25] empirically tested hypotheses on the effects of key components of consumer engagement (cognitive appraisal, affective states, participation) on consumers’ affective engagement. Zhan, Tu and Yu (2018) [26] performed a sentiment analysis on Instagram of library readers by identifying three polarities of opinion (negative, neutral and positive) and six emotions through comments (scary, loser, upset, enjoyable, happy and fun). These polarities provide new insights into understanding readers, which helps libraries provide better services. Diayanah-Abdullah and Asnira-Zolkepi (2017) [27] analyzed users’ emotions towards brands on social media, and their results show that the feeling of provocation must be managed efficiently to start interaction and a long-term relationship. Finally, Domingo, Jewitt and Kress (2015) [28] stated that, on Instagram, writing is an intrinsic part of the visual element, hence the importance of analyzing the emotional valences of the comments posted by users of the network.

Therefore, the literature found reflects the relevance that the analysis of content has had and continues to have today, in terms of the emotions it reflects and the emotional engagement that can be obtained through social networks.

### 2.3. Neuroscience at the Service of the Study of Emotions: Implicit and Explicit Measures

The measure of emotional valence is being used as an indicator of the success of social media communication campaigns. This measure is often obtained from automated emotional analyses of user comments, which are considered an explicit measure as they are self-expressed in the form of texts and emojis [7,8,22,29] (Kralj et al., 2015; Driscoll, 2015; Turnbull and Jenkins, 2016; Dubovi and Tabac, 2021). However, neuromarketing techniques can also be used to analyze the unstated (implicit) responses of the target audience. In these cases, the most common way to obtain emotional valence is through the use of technological tools that take biometric measurements, such as skin conductance or facial micro-expressions. However, neuroscientific techniques, such as electroencephalograms (EEG), can also record the measurement of emotional valence.

Although there is little empirical evidence, some studies can be found that have used neuroscience tools for the analysis of emotionality in social media. These include the study by Harris, Ciorciari and Gountas (2019) [30], which analyzes social media strategies based on action/challenge/emotion, showing the value of combining neuroscientific techniques (EEG) with traditional market research methods (psychometric survey).

Relevant studies have used a combination of measures of emotional valence to investigate different stimuli related to marketing communications. There is one study, focusing on aesthetic and utilitarian emotions in response to advertisements, that combines neuroscience research techniques (facial electromyography and skin conductance) with the study of subjective self-evaluations of emotion (Lajante et al., 2020) [31]. In the context of social media, a study has been carried out comparing two measures of emotional valence: that obtained from psychophysiological responses, and that resulting from the analysis of user comments. This study determines the existence of significant differences in unconscious and verbalized responses (Hernández-Fernández, Mora and Hernández, 2019) [32]. From the field of computer science, a study is conducted that records the physiological reactions and verbalized responses of e-game users to evaluate their experiences. Researchers recognize the potential of physiological analysis to enrich research in entertainment technology (Mandryk, Inkpen & Calvert, 2006) [33].

This shows the potential of combining neuroscientific and biometric devices with self-reported measures in the study of social networks.

### 2.4. The Use of GIFs in Social Media

GIFs have become culturally relevant in the digital context, especially in social media. They are a good tool for sensory appeal through the use of movement, color and repetition (Ash, 2015) [34]. They are considered a suitable resource to generate emotionality, as they can represent a wide variety of feelings (Bourlai and Herring, 2014) [4]. In addition, due to their simplicity and high number of meanings, they manage to arouse empathy with the content shown (Miltner and Highfield, 2017) [35]. These qualities lead brands to use GIFs to design experiences with affective qualities (Gürsimsek, 2016). [36]

Previous studies have investigated the use of GIFs in social media, showing interesting results. According to one of them, GIFs are the most attractive resource on Tumblr in terms of likes and reblogs (Bakhshi et al., 2016) [5]. Another study stated that the object that appears and the associated emotions are more important than the movement. It also concluded that the interest generated is associated with the number of likes the GIF receives, but does not correlate with reblogging it (Gygli and Soleymani, 2016) [6]. In fact, there is an interesting line of research that focuses on designing an affective computing tool for the automated analysis of the emotions represented in GIFs based on the facial expressions they contain (Brendan, Bhattacharya, and Chang, 2014; Chen, Rudovic and Picard, 2017) [37], [38]. Finally, Rua-Hidalgo et al. (2021) [39] conducted a two-phase study on GIFs used by commercial brands. In the first phase, they combined the biometric tools of automated observation of facial expressions, skin conductance and eye position to observe the emotional state that GIFs of well-known brands cause in participants. Furthermore, they compared them with the effects caused by static images of the same brands, concluding that GIFs achieve user engagement and cause a “state of well-being and pleasure” (Russell’s Circumplex Model, 1980) [40]. In the second phase, they used the implicit association test to observe unconscious associations related to well-known brands, and the results obtained show that participants believed that well-known brands are quality brands. The correlation found between the results of the two studies reveals that GIFs, while arousing positive emotions and leading to engagement, do not achieve an enthusiastic state when brands are internalized as quality brands.

In this way, GIFs are an attractive option when it comes to generating emotions, which can translate into higher conversions on social networks. For this reason, addressing how they can be effective in communicative terms, combining explicit and implicit measures can provide information that has not been explored so far.

## 3. Research Questions and Hypotheses

The following are the research questions and hypotheses derived from previous literature.

**RQ1.**  Are there differences between implicit and explicit measures of emotional valence in Instagram users?

**Hypothesis** **1.** *The measure of explicit valence of Instagram users’ response to GIFs will be more positive than the measure of implicit valence.*

**RQ2.**  What might account for these possible differences?

**Hypothesis** **2.** *The greater the user engagement with GIFs, the smaller the difference between implicit and explicit measures of emotional valence.*

**Hypothesis** **3.** *The longer the comment, the smaller the difference between implicit and explicit measures of emotional valence.*

**Hypothesis** **4.** *The greater the proportion of emojis in the comments on GIFs, the greater the difference between implicit and explicit measures of emotional valence.*

**RQ3.**  Are explicit comments and biometric tools equally effective in identifying the emotions felt by subjects?

**Hypothesis** **5.** *Biometric tools are more accurate predictors for assessing the basic type of emotion that is aroused in response to GIFs.*

**Figure 1:**
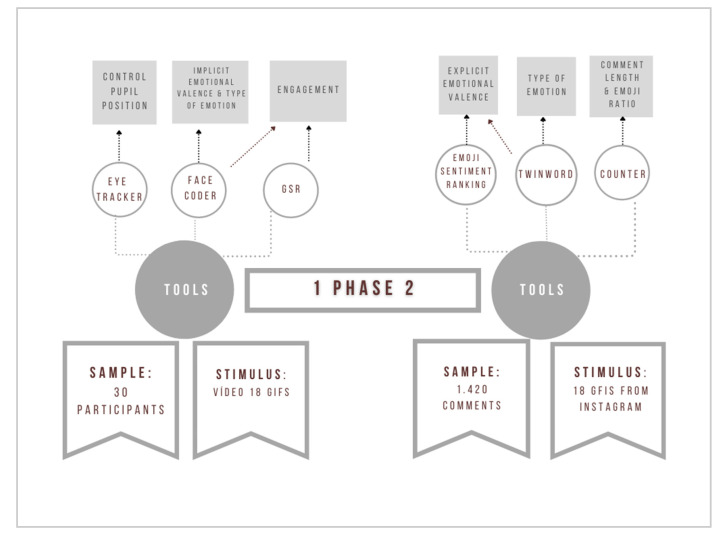
Visualization of the methodology of phases 1 and 2.

In order to test these hypotheses, this research was divided into two phases focused on inferring the emotional valence derived from a selection of GIFs posted on Instagram (Figure 1). In the first phase, techniques from consumer neuroscience or neuromarketing were applied to obtain a measure of implicit emotional valence and engagement. The second phase complemented the first by analyzing the explicit emotional valence based on the semantic analysis of the comments on each GIF. Finally, a comparison was made between the emotional data obtained implicitly and explicitly.

## 4. Materials, Methods and Results

### 4.1. Phase 1. Experimental Study of Neuromarketing Applied to GIFs

In this first phase, neuromarketing devices (face coder, GSR and eye tracker) are applied to analyze and quantify the emotional valence, the engagement generated and the type of basic emotion caused by 18 Instagram GIFs selected for being used by renowned brands and for having a high number of likes. The selected GIFs are images in movement with an approximate duration of 4 s. All the images are high-quality, some taken outdoors and others indoors. Concerning audiovisual treatment, some contain a filmed scene and others constantly repeat a moving image. The visual contents are varied: people, objects and animals, both in the foreground and in the background.

The valence variable allows us to identify the sign of the emotion (positive or negative). The engagement variable indicates the emotional state the person is in when viewing the stimulus, and is extracted through the combination of emotional valence and activation. The type of basic emotion recorded indicates which specific emotions have been experienced through the recording of facial expressions (happiness, surprise, anger, disgust, fear and sadness).

The three variables provide the measure for each subject and each GIF, and these individual data are added to the aggregate values for the group, thus obtaining a quantitative value for each variable for each GIF.

#### 4.1.1. Participants

Participants were selected randomly, with the inclusion criterion being regular use of at least one social network.

The sample size was 30 participants. This sample size was considered representative with a probability of error of less than 1% [41,42,43] (Cohen, 1992; Sands, 2009; Hensel et al., 2017). The size is adequate to provide sufficient knowledge about the stimulus for the purpose of the research. Furthermore, the distribution of the sample was based on the Interactive Advertising Bureau and the Elogia study (2018) [44], which described the profiles of social network users. Therefore, the composition was 33% for each age range (16–30; 31–45; 46–55), with 53% women and 47% men.

#### 4.1.2. Stimuli

The stimulus used in this experimental phase was a video of 2 min and 26 s, made from a random combination of 18 GIFs, incorporating distractors between each of them. In order to avoid presentation bias, three different videos were edited with the elements randomly ordered.

The selection of the GIFs was based on a review of all GIFs posted on Instagram (1 March–10 April 2019) by the top 100 international brands (2017 Best Global Brands ranking—Interbrand, 2017 [45]). The 18 GIFs with the highest number of likes were selected.

All subjects viewed one of the three versions of the video. During the presentation of the stimulus, facial micro-expressions, skin conductance level and pupil direction were recorded.

#### 4.1.3. Devices

The GIFs were presented via a laptop (Windows 10 operating system) and a 24-inch monitor. In addition, an external webcam was used to record facial expressions and pupil direction.

#### 4.1.4. Measuring Tools

*Face coder.* This records the data from the decoding of the person’s face through software that analyzes the image provided by the webcam. The software developed by the company INTERACTÚA+ was used.

The Face Coder tool provided six concrete emotions (joy, surprise, sadness, fear, anger and disgust), with their respective value, recorded from facial micro-expressions that were generated in the presence of each GIF. Subsequently, the values of these emotions registered in all participants were aggregated, obtaining a single value for each emotion per GIF.

Previous research in communication and marketing stimuli using this tool for the analysis of emotion type has been considered as the empirical background (McDuff, El Kaliouby and Picard, 2012; Bellman, Wooley and Varan, 2016; Goyal and Singh, 2018; Mundel et al., 2018) [46,47,48,49].

*Galvanic Skin Response (GSR).* This records skin conductance levels through electrodes placed on the fingers of the subject’s hand. The eSense^®^ device was used.

Previous studies involving GSR to analyze unconscious responses to marketing stimuli were taken into account in the design (Weibel et al., 2019; Walla, Koller, Brenner and Bosshard, 2017; Guerreiro, Rita and Trigueiros, 2015; Reimann, Castano, Zaichkowsky and Bechara; 2012) [50,51,52,53].

*Eye-tracker.* This has been included in the experimental study to ensure that the participant is viewing the stimulus. It consists of a device that tracks the eye and records the subject’s gaze while viewing a stimulus.

#### 4.1.5. Data Analysis

All recorded data were first processed in Excel in order to clean them and make them manageable in the SPSS statistical software.

Specifically, the data recorded by the facial emotion identification tool (face coder) determine the valence of the emotions (positive or negative) and the type of emotion according to the classification of the six basic universal emotions (Ekman, 1993) [14]. The recording of the level of skin conductance (GSR) during the visualization of the stimulus was used to obtain the level of arousal. From these two records, the engagement variable was calculated, which indicates the emotional state in which the participants found themselves while they were watching the video, as it combines the type of emotion with the intensity of this emotion. In addition, as a control measure, the position of the pupil (eye tracking) was monitored, which made it possible to verify the stimulus that was really generating the emotion.

Emotional valence and engagement were tested for normality and homogeneity of variances. The basic emotion type was quantified to get an approximation of how much each emotion was experienced upon exposure to each GIF.

#### 4.1.6. Ethical Issues

The study was approved on 20 April 2018 by the Ethics Committee of the Universidad Internacional de la Rioja, Spain, under the number LABNMKT-001-2018.

The ethical protocols of the World Medical Association Declaration of Helsinki (1964) were followed. In addition, informed consent was obtained from all participants, assuring data confidentiality.

#### 4.1.7. Procedure

After signing the informed consent form, each participant was placed in front of the computer. Both the face coder and the GSR were checked to ensure that they were calibrated and working correctly, and the baseline measurement was taken. Then, one of the three videos with the 18 stimuli was randomly played. Once the video had finished, they were thanked for their participation and accompanied to the exit. Each test lasted about 6 min in total.

In order to obtain the baseline of the GSR tool, sensors were placed on the non-dominant hand of the participants in such a way that it began to collect values prior to the presentation of the stimulus, thus establishing the skin conductance baseline of each participant. At the same time, the computer equipment and the tool software were synchronized. On the other hand, the eye tracker and Face Coder tools shared the same software and implementation. The first step was to regulate the webcam to achieve optimal lighting conditions and get the face of the participant centered on the screen. Then, the calibration software allowed us to obtain the baseline. This involved following a point that moved across the screen and picking up the point where the pupil is located.

#### 4.1.8. Results

The Shapiro–Wilk normality test (see Table 1) shows that the variables implicit emotional valence (V_IE_) and engagement (Eg) are close to normal (V_IE_: *p* = 0.779 > 0.050; Eg: *p* = 0.772 > 0.050). Furthermore, their variances are homogeneous, according to the results obtained in Levene’s test of equality of variances (V_IE:_ *p* = 0.873 > 0.050; Eg: *p* = 0.818 > 0.050). The measures of the variables analyzed are shown in Table 1.

The 18 GIFs selected reflect the six basic emotions. The emotion that appears most frequently is sadness, followed by anger, as shown in Table 2.

### 4.2. Phase 2. Sentiment Analysis of GIF Content on Instagram

In this phase, a quantitative methodology was implemented and a content analysis was carried out, using data mining and sentiment analysis. To do this, the comments on the Instagram GIFs selected in Phase 1 were obtained.

The explicit emotional valence of the comments was quantified (negative or positive), both the one derived from the text and the one derived from the emojis in the comments (negative and positive), as well as the length of the comment itself (including both text and emojis) and the proportion of emojis in the comments.

#### 4.2.1. Stimuli

A total of 1420 comments were analyzed. These came from each of the 18 GIFs used in Phase 1. Up to 100 comments were extracted from each GIF (15 April–1 May 2021). If they had less than 100 comments, all existing comments were extracted. Similarly, if they had more than 100 comments, only the first 100 were used.

#### 4.2.2. Measuring Tools

*Sentiment analysis of comments*. To obtain information on the length and composition of the comments, a program written in Python 3 was used. This program takes as input an excel document, generated through the Export Comments application, with the comments on each GIF (the openpyxl library was used). The comments were cleaned up using a function that removes strange punctuation symbols and tags that correspond to other users. The Emoji library was used to identify the emojis present in the comments. Then, a function was created to obtain the number of words, the average lengths of the comments, and the percentage of words and emojis in the comment. With all the information obtained, a new Excel document was generated (using openpyxl) that includes the following statistics: average number of words in the comments, average number of emojis and percentage of emojis versus words for each GIF.

The Twinword API (https://www.twinword.com/; accessed on 31 May 2021) was used. This natural language processing (NLP) interface, based on the Python programming language, detects the intentionality of sentences and paragraphs. The measure it provides is the overall score of the analyzed text. Thus, values lower than −0.05 are considered negative, values higher than 0.05 are considered positive, and all values in between are considered neutral. In addition, thanks to this application, a list of the most emotionally charged words and adjectives was obtained from the comments.

*Detection of the basic emotions in the comments*. Through Twinword’s Emotion Analysis API, the basic emotions in each of the 18 GIFs were identified.

*Analysis of the emotionality of emojis*. The emotional valence of emojis was obtained using the Emoji Sentiment Ranking (http://kt.ijs.si/data/Emoji_sentiment_ranking/; accessed on 31 May 2021), developed and validated by Kralj et al. (2015) [29]. 

*Content analysis*. We developed our own counting application to obtain the maximum length of the comments, as well as their composition (proportion of text and emojis).

#### 4.2.3. Procedure and Data Analysis

(1) Compilation of comments on each GIF

Using the URLs of the Instagram GIFs, up to 100 comments were collected for each of the 18 GIFs under study. Using Export Comments (https://exportcomments.com/; accessed on 31 May 2021), an Excel file was obtained for each GIF. If it had more than 100 comments, the tool extracted 100; if this number was lower, all comments were extracted.

Subsequently, all comments were translated into English as the sentiment analysis tool only evaluates content in this language. This was done via Google Translator (https://translate.google.com/?hl=fr&tab=TT; accessed on 31 May 2021).

(2) Analysis of the explicit emotionality of each GIF and calculation of the variable V_EE_ (Table 3)

The analysis of the emotionality of the textual content was carried out with Twinword. An emotion value was obtained for each comment in the GIF, calculated by averaging the total emotion value of each GIF, which was in the range −1 to 1.

The emotional valence of the emojis was then obtained by means of the Emoji Sentiment Ranking.

In order to obtain one single standardized value for the explicit valence measure, all text and emoji values were taken together and divided by the number of comments. This resulted in the variable Explicit Emotional Valence (V_EE_).

(3) Analysis of the Composition of Comments and Calculation of Variables LgC and P_emj_ (Table 3)

In order to count the length (LgC) and the proportion of emojis in the total content of the comments (P_emj_), we used our own counting application, designed in Python language. Thus, we obtained the average value of the length of the comments in each GIF and the percentage of emojis used in their comments.

(4) Analysis of the Differences between Explicit and Implicit Emotionality and Calculation of the Variable VD (Table 3)

The variable Emotional Valence Difference (EVD) was obtained by the difference between the values recorded explicitly (comments) and implicitly (biometric tools):

VD = V_EE_ − V_IE_

#### 4.2.4. Results

##### Implicit Measure of Valence Versus Explicit Measure of Valence

The first step was to calculate normality for the V_IE_ and V_EE_ through the Shapiro–Wilk test (Table 4). The distributions were found to approach normality for both V_IE_ and V_EE_ (*p* > 0.050). In addition, the assumptions of homogeneity of variance were met using Levene’s test for equality of variances (*p* = 0.873 > 0.05).

Since the normality and homoscedasticity criteria were met, a t-test for independent samples was performed to compare the average of implicit valence and explicit valence. The results, as shown in Table 5, reveal statistically significant differences between V_IE_ (MV_IE_ = 0.148, SD = 0.036) and V_EE_ (MV_EE_ = 0.407, SD = 0.030; *t*(34) = −7.178, *p* = 0.000). Therefore, emotional valence was significantly higher when users expressed themselves through comments on Instagram than when the emotional valence aroused by GIFs was recorded through biometric tools. Consequently, H1 is confirmed. Moreover, in both cases the average emotional valence was positive.

##### Differences between Implicit and Explicit Measures of Emotional Valence for Each GIF

A new continuous variable is defined: the difference of the emotional valence between the implicit and the declared measures in each GIF (VD). This value was calculated for each GIF. Additionally, in order to obtain which variables could explain this difference between the two measures of valence, different correlations between different variables were studied through Pearson’s bivariate correlation coefficient. Specifically, these variables were engagement (Eg), comment length (LgC) and proportion of emojis in the comments of the GIFs (P_emj_).

(1) Relation between Valence Difference (VD) and Engagement (Eg)

In the relation between VD and Eg, the correlation showed a statistically significant inverse relation with a negative sign, as shown in Table 6 (*r* = −0.546, *p* > 0.050). This indicates that the more engagement a GIF generates, the smaller the difference in emotional valence measured explicitly (through comments) and implicitly (biometric tools), thus confirming H2.

(2) Relation between Valence Difference (VD) and Comment Length (LgC)

A possible significant and positive relation between VD and LgC has been hypothesized. However, the results indicate that there was no statistically significant relation between VD and LgC (*r* = −0.380, *p =* 0.120), rejecting H3. Therefore, the greater difference found between the psychophysiological and the stated measure of valence was not due to the length of the comment.

(3) Relation between the Valence Difference (VD) and the Proportion of Emojis (P_emj_)

With respect to the relationship between VD and P_emj_, as hypothesized, a significant and positive correlation was found (*r* = 0.631, *p* > 0.010), as shown in Table 7. This indicates that the greater the proportion of emojis used in comments, the greater the difference that will be found between the psychophysiological and the stated measure of valence. Therefore, H4 was accepted.

Comparison between the Emotions Generated by the GIFs According to Whether They Were Measured Implicitly or Explicitly.

In order to find out which type of measurement is more effective as a predictor, understanding effectiveness in this case in terms of precision, the specific emotions that gave rise to both implicit and explicit emotional valence were obtained (see Table 8).

**Figure 2:**
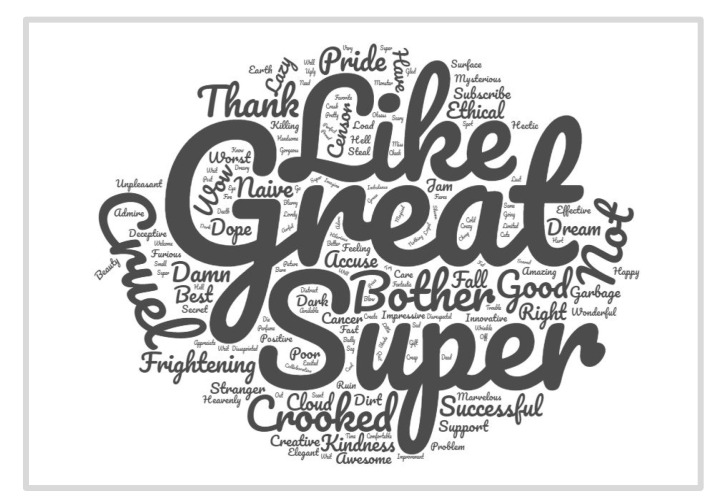
Visualization of the most emotionally charged nouns and adjectives extracted from the totality of the GIF comments.

As a result, while the biometric tools detected the six basic emotions (happiness, surprise, anger, disgust, fear and sadness) in the 18 selected GIFs, when analyzing the comments, it was not possible to detect all the emotions. Only the emotion happiness appeared in all 18 GIFs, while more negative emotions such as anger or disgust were not detected in any of them. In fact, most of the words used in the comments alluded to positivity (see Figure 2). Given that the biometric tools were more accurate in detecting the six emotions, we accept H5.

## 5. Discussion

The present study reflects the emotional impact that can be achieved through the use of GIFs in social networks [4,35,36], (Bourlai and Herring, 2014; Gürsimsek, 2016; Miltner and Highfield, 2017), specifically Instagram. Moreover, the use of different implicit and explicit techniques in the two phases of the study highlights the need to use biometric and self-reported tools in a complementary manner [31,32] (Hernández-Fernández, Mora and Hernández, 2019; Lajante et al., 2020) in order to eliminate biases—biases that undoubtedly occur if only the analysis of the comments declared by the subjects is taken into account.

By comparing implicit and explicit emotional valence, we find that social network users tend to express comments with a significantly higher (more positive) emotional charge than what they actually feel (before processing the stimulus rationally). Biometric tools can therefore help to provide a more accurate analysis.

This finding raises the question of what might account for the differences between the two valence measurements. Thus, the possible influences of engagement, the length of the comments, or the higher proportion of GIFs on the difference in valence are investigated.

As for the relation between the differences in valence and engagement, a significant inverse relation is evident. This indicates that the greater the engagement with the GIF, the smaller the difference between the valences. This relation can probably be explained by understanding engagement as commitment to the brand. This commitment could mean that users are less vulnerable to external influences, so that the emotions expressed would be more in line with what the subjects really feel towards the brand. In contrast, with a lower level of engagement, the emotions declared are under greater external influence: conditioning due to being a quality brand [39] (Rúa-Hidalgo et al., 2021), a sense of belonging, prestige, etc. In short, well-known brands need to surprise in order to activate users; when this does not happen, less engagement is generated (Rúa et al., 2021) [39]. However, it remains possible that the comments on social networks continue to be positive, moving even further away from the emotion that is really felt.

On the other hand, the length of the comment is not found to influence the differences between implicit and explicit measures of valence. This suggests that the emotionality of the words contained in the comment matters more than the length of the comment itself.

The proportion of emojis has not been found to be a reliable predictor when assessing the emotional level of the social network user. A higher proportion of emojis in comments generates a greater distortion between implicitly and explicitly measured valence. This could be due to the fact that text provides greater possibilities for expressing emotions felt (greater semantic richness), while with emojis the range of expression is smaller, and more limited in its emotional gradation.

Finally, in the comparison of the type of emotion recorded by biometric tools and in the declared comments, a higher number of emotions were detected when recording data with neuromarketing devices.

We suggest that, when there is public exposure, through comments on social networks users express more positive emotions (happiness and surprise), avoiding more negative ones such as anger or disgust [17,18] (Reinecke and Trepte, 2014; Waterloo, Baumgartner, Peter and Valkenburg, 2018). It is possible that the external influences to which we as social beings are subjected (need to belong, social judgement, status, etc.) are the cause. However, these influences disappear when the implicit emotion is recorded through neuromarketing tools, making it possible to identify a greater and more precise variety of emotions.

All of the above indicates that using the comments received on social networks as the only indicator of the level of success of a communication action can lead to a false sense of success for well-known brands.

It also shows that the implicit measurement of emotion is more effective, both in quantifying the emotional level of subjects and in identifying the basic type of emotion that subjects experience, thus achieving greater richness and precision in the analysis.

## 6. Implications of the Work

Biometric tools have proven to be very useful in specifying and settling the emotions perceived by users. This does not mean eliminating other traditional methods of analysis, but rather using them complementarily. In short, they highlight what the users are not even able to identify themselves.

## 7. Limitations and Future Lines of Research

A limitation of the research lies in the use of Google Translator to transcribe sentiment analysis comments into English. This basic translation tool can cause the context to be lost in translation; however, less than 11% of the comments needed to be translated, and in most cases, they were short, easily translated comments.

In addition, as the GIFs were always from well-known brands, the level of emotionality in the comments may be different from if they were used in the context of non-known brands.

It would be interesting to carry out future research to study whether the use of other types of stimuli in social networks (stories, photos or videos) can achieve similar results to those achieved with GIFs.

## Figures and Tables

**Table 1:** Statistic results of the variables in Phase 1.

Variables	Media	Standard Deviation	Standard Error
Implicit emotional valence (V_IE_)	0.1480	0.0356	0.0084
Engagement (Eg)	0.0025	0.0309	0.0073

**Table 2:** Types of emotions registered in each GIF by the face coder.

Links ^1^	Happiness	Surprise	Anger	Disgust	Fear	Sadness
GIF1	0.0307	0.005	0.0914	0.0487	0.0097	0.1062
GIF2	0.0579	0.005	0.0903	0.0310	0.0119	0.1499
GIF3	0.0475	0.0121	0.0852	0.0193	0.0198	0.1304
GIF4	0.0414	0.0042	0.083	0.0272	0.0059	0.1455
GIF5	0.029	0.047	0.0793	0.0092	0.0162	0.1263
GIF6	0.0415	0.0536	0.0827	0.0169	0.0093	0.1242
GIF7	0.0459	0.0705	0.0649	0.0121	0.0053	0.123
GIF8	0.0519	0.0628	0.0977	0.0200	0.0096	0.1499
GIF9	0.0371	0.0059	0.0628	0.0391	0.0078	0.1013
GIF10	0.0528	0.0008	0.0802	0.0238	0.0076	0.1395
GIF11	0.0398	0.0665	0.0722	0.0206	0.0322	0.1245
GIF12	0.0409	0.0383	0.0800	0.0242	0.0097	0.1415
GIF13	0.0576	0.0027	0.0506	0.0189	0.0056	0.1445
GIF14	0.0455	0.0029	0.0884	0.0428	0.0039	0.1305
GIF15	0.0353	0.0129	0.0778	0.0361	0.0086	0.1136
GIF16	0.0394	0.0343	0.0644	0.0083	0.0069	0.1248
GIF17	0.0184	0.0328	0.0817	0.0290	0.0366	0.1396
GIF18	0.0422	0.0152	0.1021	0.0347	0.0082	0.1379
Media	**0.0419**	**0.0263**	**0.0797**	**0.0257**	**0.0119**	**0.1307**

^1^ Links to GIFs for inclusion in Table 2 were obtained on 15 May 2021.

**Table 3:** Variables, dimensions and tools of Phases 1 and 2.

Variable	Dimension	Tool
Implicit Emotional Valence (V_IE_)	Value of emotion	Face coder
Engagement (Eg)	Emotional state	Face coder + GSR
Explicit Emotional Valence (V_EE_)	Value of emotion	Twinword
Difference V_IE_ y V_EE_ (VD)	Difference between valences	V_EE_−V_IE_
Comment length (LgC)	Number of elements that appear in the comment	Element Counter (own design)
Proportion of Emojis (P_emj_)	Percentage of emojis over total number of elements in a comment	Element Counter (own design)
Type of basic emotion	Identification of basic emotions	Twinword Emotion Analysis API (explicit method) Face coder (implicit method)

**Table 4:** Result for normality test.

	Shapiro–Wilk	
Data Origin	Statistic	Sig.
Biometric tool (V_IE_)	0.969	0.779
Instagram comments (V_EE_)	0.943	0.323

The correlation was significant at the 0.05 level (bilateral).

**Table 5:** Statistical results of emotional valences.

Data Origin	Average	Standard Deviation	Standard Error
Biometric tool (V_IE_)	0.1480	0.0356	0.0084
Instagram comments (V_EE_)	0.4068	0.0305	0.0072

**Table 6:** Correlation between the variable Implicit–Explicit Valence Difference and Engagement.

	Engagement (Eg)	Implicit-Explicit Valence Difference (VD)
Engagement (Eg)	1	−0.546 *
		0.019
Implicit–Explicit Valence Difference (VD)	−0.546 *	1
	0.019	

* The correlation is significant at the 0.05 level (bilateral).

**Table 7:** Correlation between the variable Explicit–Implicit Valence Difference and the percentage of emojis in the comment.

		Percentage of Emojis in the Comment (Pemj)	Explicit–Implicit Valence Difference (VD)
**Percentage of emojis in the comment (Pemj)**	Pearson’s correlation	1	0.631 *
	Sig. (bilateral)		0.005
**Explicit–Implicit Valence Difference (VD)**	Pearson’s correlation	0.631 *	1
	Sig. (bilateral)	0.005	

* The correlation was significant at the 0.01 level (bilateral).

**Table 8:** Number of GIFs containing each type of emotion by measurement.

	Happiness	Surprise	Anger	Disgust	Fear	Sadness
Biometric tool	18	18	18	18	18	18
Instagram comments	18	12	0	0	6	1

## Data Availability

Table 2 provides the links to the publicly archived data analysed in the study (last acces on 15 May 2021). GIF1; GIF2; GIF3; GIF4; GIF5; GIF6; GIF7; GIF8; GIF9; GIF10; GIF11; GIF12; GIF13; GIF14; GIF15; GIF16.
